# Opening the doors for spillovers: a contingency view of the effects of work from home on the work–home interface

**DOI:** 10.3389/fpsyg.2023.1191657

**Published:** 2023-07-05

**Authors:** Felix Bölingen, Alejandro Hermida Carrillo, Ingo Weller

**Affiliations:** LMU Munich School of Management, Munich, Germany

**Keywords:** work–home interface, job satisfaction, relationship satisfaction, work from home, technology

## Abstract

Why do employees experience work from home (WFH) differently? We draw on boundary theory to explain how WFH influences employees’ work–home interface. WFH intensity increases negative spillovers (i.e., work-to-home conflict and home-to-work conflict) and positive spillovers (i.e., work-to-home enrichment and home-to-work enrichment) between the work and home domains. Negative spillovers can be mitigated through high-quality work equipment and beneficial spatial conditions at home. Domain centrality predicts who can benefit from increased WFH intensity. We test our theory with a sample of 545 employees, obtained through a two-step random sampling procedure in the city of Munich/Germany during the COVID-19 pandemic. We find that WFH intensity increases work-to-home conflict and home-to-work enrichment, affecting employees’ relationship satisfaction and job satisfaction. High-quality work equipment mitigates the detrimental effects of WFH. Employees with a high family centrality can reap benefits of more WFH because they experience more home-to-work enrichment. The simultaneous desirable and detrimental effects of WFH intensity can partly explain why studies have found heterogenous WFH experiences among employees.

## 1. Introduction

The COVID-19 pandemic has renewed scholarly interest in the effects of work from home^1^. Interestingly, studies have documented both positive and negative effects of WFH on employees’ work and home lives (Allen et al., 2014; Kniffin et al., 2021; Massar et al., 2023), but have failed to provide a comprehensive explanation of why employees experience WFH differently. For example, employees who work from home report difficulty mentally unplugging during breaks and downtime, or being interrupted by their partners (Dunn, 2020). However, employees also report that WFH allows them to spend breaks with loved ones and return to work with renewed energy (Gohmann, 2020). Related research suggests that spillovers at the work-home interface may be the mechanism explaining why employees experience WFH differently (Huyghebaert-Zouaghi et al., 2022).

**Figure 1:**
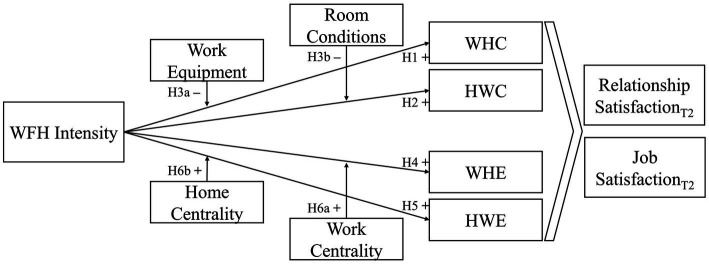
Theoretical model.

The work-home interface encompasses four types of inter-role spillovers, based on the directionality (work-to-home and home-to-work) and quality (conflict vs. enrichment) of the interaction: work-to-home conflict and home-to-work conflict are negative spillovers, and work-to-home enrichment and home-to-work enrichment are positive spillovers. Although the four spillovers are theoretically and empirically distinct, they share some common predictors (Frone, 2003; Greenhaus and Powell, 2006; Huyghebaert-Zouaghi et al., 2022). Building on boundary theory (Ashforth et al., 2000), we first theorize that WFH intensity—which is the number of hours worked from home—can trigger all four types of inter-role spillovers. This view implies that WFH intensity has positive and negative effects on the work-home interface as well as positive and negative downstream consequences. Second, we develop a contingency view to examine the role of situational and personal moderators: work equipment, room conditions, and domain centrality. Specifically, we propose that the quality of one’s work equipment can mitigate the work-to-home conflict, whereas room conditions can mitigate the home-to-work conflict. In addition, we propose that the centrality of the work and home domains can enhance the relationships between WFH and enrichment in the respective other domain. Third, we build on the domain specificity (Frone et al., 1992) and source attribution models (e.g., Shockley and Singla, 2011) to examine the downstream influences of spillovers on employees’ satisfaction with their romantic relationships and jobs. Figure 1 depicts our theoretical model.

To test our theory, we use a unique longitudinal data set that we collected in a two-step random sampling procedure during the COVID-19 pandemic in the city of Munich/Germany. We test our hypotheses with moderated regression analyses and use multiple mediation models to analyze whether negative and positive spillovers exist in parallel, and what their individual and joint effects on satisfaction outcomes are.

Our work contributes to the literature in three ways. First, we theorize and test how WFH intensity affects all four dimensions of the work-home interface. WFH literature—before (for a review see Allen et al., 2015) and during (e.g., Kossek et al., 2021; Leroy et al., 2021) the pandemic—has primarily studied negative spillovers, whereas positive spillovers have received only scattered mentions (e.g., Rothbard et al., 2021) and less empirical scrutiny (e.g., McNall et al., 2009; Huyghebaert-Zouaghi et al., 2022). The joint consideration of negative and positive spillovers has theoretical and practical relevance. Theoretically, boundary theory predicts that WFH intensity can be a stressor and a resource simultaneously, triggering negative and positive spillovers through role integration (e.g., Ilies et al., 2009; Kossek et al., 2012; McNall et al., 2015). A one-sided focus on negative spillovers does not do justice to the complexity of the theory and might lead to incomplete practical considerations.

Second, we test whether domain centrality moderates the relationships between WFH intensity and spillovers, thus contributing to an ongoing debate in boundary theory (Capitano and Greenhaus, 2018; Lapierre et al., 2018), which has competing views on whether domain centrality is more strongly related to positive spillovers in the receiving domain *or* in the originating domain. Our focus on workplace characteristics as moderators further provides actionable insights about how the negative consequences of WFH can be mitigated through organizational interventions (Kniffin et al., 2021).

Finally, we theorize and test how WFH intensity affects the satisfaction of employees with their jobs *and* their romantic relationships, an important home-related outcome. So far, the effects of WFH on the private lives of employees are rather unknown, both to research and organizations. By examining employees’ relationship satisfaction, we respond to calls to study WFH outcomes that are important but not directly relevant for the profits of organizations (Kramer and Kramer, 2021).

## 2. Theoretical background and hypotheses

In research and practice, boundary theory (Nippert-Eng, 1996; Ashforth et al., 2000; Clark, 2000) has been widely used to understand the dynamics of the work and non-work domains as well as their interactions. Specifically, boundary theory posits that roles are surrounded by psychological, physical, and temporal lines of demarcation (i.e., boundaries). Individuals navigate the boundaries and manage the transitions and interactions between the roles and domains to maintain a balance between the work and non-work domains. We use boundary theory as the overarching theoretical framework of our research. In what follows, we first explore how and why WFH can blur the boundaries between the work and the home domains, with negative consequences for employees’ work-home interface and their work and home lives.

### 2.1. Work from home intensity and negative spillovers

Work from home requires employees to integrate their work and home roles, because they engage in work activities in physical spaces that are normally dedicated to their private lives. In such a situation, work roles are necessarily—and often unintentionally—enacted during times that are normally devoted to the home domain, and vice versa. This is embodied in the concept of boundary control (Kossek et al., 2012; Lapierre et al., 2016). With decreased control over one’s boundaries, interruptions from partners, children, and other household members are more likely and can detract employees from work (Leroy et al., 2021), just as home domain activities (e.g., household duties, homeschooling) are more likely to interfere with one’s work (Shockley and Allen, 2015), resulting in home-to-work conflict.

Employees engaged in WFH can also experience work-to-home conflict. The absence of face-to-face communications in the office can foster an “always connected” work culture. With an abundance of digital technologies and communication tools available, role transitions take place faster and more frequently, which in turn increases the likelihood of employees engaging in work activities during times that were traditionally dedicated to the home domain (Ferguson et al., 2016). In line with this logic, Lapierre et al. (2016) found that mandatory WFH was positively related to work-to-home conflict in a sample of financial sales professionals, and Leroy et al. (2021) reported that, since the onset of the pandemic, the number of work and non-work interruptions has increased, which applies equally to work-to-home conflict and home-to-work conflict.

Therefore, WFH intensity—defined and operationalized as the number of hours worked from home—can create negative spillovers. WFH intensity will often lead to a stronger WFH concentration, such that single (or multiple) days of the week are completely devoted to WFH. Whereas there are some obvious advantages (which we discuss below) of intensive WFH, it comes along with a loss of boundary control. The loss of boundary control increases both the potential of work-to-home conflict and home-to-work conflict. For example, when complete days are worked from home, employees might find it increasingly difficult to switch off mentally after work, such that their non-work relationships can suffer. Likewise, with more hours worked from home it becomes increasingly difficult to avoid the regularly occurring interruptions of the home domain, such as children leaving to and returning from school, or household duties like cooking or house cleaning. Consequently, we pose the following hypotheses:

*Hypothesis* 1: WFH intensity increases work-to-home conflict.

*Hypothesis* 2: WFH intensity increases home-to-work conflict.

### 2.2. Boundary conditions of the WFH intensity—negative spillover relationships

Although it carries the potential for negative spillovers, WFH intensity is neither necessarily bad for everyone nor under all circumstances. In fact, meta-analyses prior to the pandemic have reported a small *negative* effect of WFH intensity on negative spillovers (Gajendran and Harrison, 2007; Allen et al., 2013). One plausible explanation is self-selection. Most employees who self-selected into WFH prior to the pandemic saw primarily the advantages of it, whereas many employees who worked (or had to work) from home during the pandemic adapted to the new circumstances only gradually and suboptimally. A critical factor determining whether employees see primarily the upsides or downsides of WFH are their working conditions at home, including work equipment (e.g., laptop, broadband infrastructure) and spatial situation (e.g., a separate office room at home).

If employees use low-quality work equipment at home (e.g., a couple sharing the same computer or a slow internet connection), this is likely to impede the proper execution of work tasks and can trigger stressful reactions (Chong et al., 2020; Wang et al., 2020). With increasing WFH intensity, stress and negative emotions are becoming more likely, leading to more negative spillovers to the home domain (e.g., Vaziri et al., 2020). In contrast, high-quality work equipment at home (e.g., an own laptop, a fast broadband internet connection) facilitates employees’ job activities so that they can be performed smoothly and seamlessly, which mitigates negative spillovers. We propose:

*Hypothesis* 3a: The quality of one’s work equipment at home moderates the positive effect of WFH intensity on work-to-home conflict, such that the effect is attenuated at high levels of work equipment quality (relative to low levels).

A separate office room at home allows individuals to spatially separate from the home sphere, which is a common boundary management strategy (Kreiner et al., 2009; Fonner and Stache, 2012). When employees retreat to this dedicated workspace, a boundary is drawn and interruptions from the home domain are mitigated or completely avoided (Sonnentag et al., 2010; Allen et al., 2021; Bundeskriminalamt, 2021). We hypothesize:

*Hypothesis* 3b: The spatial situation at home moderates the positive effect of WFH intensity on home-to-work conflict, such that the effect is attenuated when employees have a separate home office room (relative to not having one).

### 2.3. Work from home intensity and positive spillovers

More recent developments in the boundary theory field have highlighted that role integration can also facilitate positive spillovers (e.g., Ilies et al., 2009; Kossek et al., 2012; McNall et al., 2015). For example, Ilies et al. (2009, p. 100) note that “by integrating work and family domains employees can magnify the benefits of the positive features of work.” We underline this notion by arguing that WFH intensity can also have positive spillover effects, for two main reasons.

Just as WFH intensity increases the probability of interruptions of one’s work tasks at home, we argue that it can also increase the frequency of positive resource transmissions between the work and home domains. Some of the interruptions that can cause conflict can actually also provide respite from taxing roles, as suggested by the micro-break literature (Bennett et al., 2020) and found by Wu et al. (2021). Micro-breaks can be used to solve pressing issues that would otherwise lead to mental absence (Ashforth et al., 2000; Kossek et al., 2012). In addition, when employees are working from home, they and their loved ones can notice cues of challenging work-related situations and can help each other (e.g., provide emotional support). Giving and receiving care feels good (Feeney and Collins, 2001) and creates positive emotions that can spill over from one domain to the other.

WFH intensity can also increase the intensity of positive spillovers. Face-to-face interactions transmit affect and information more immediately and effectively than digital, text-based interactions or phone calls (Judge and Ilies, 2004; Schiffrin et al., 2010). Positive experiences from an enjoyable lunch with one’s significant other (Bosch et al., 2018) remain accessible during a work meeting when this takes place in the next room rather than miles and hours away. Similarly, good experiences from work remain fresh and pure when communicated immediately to an accessible partner who can reinforce the positive experience. We hypothesize:

*Hypothesis* 4: WFH intensity increases work-to-home enrichment.

*Hypothesis* 5: WFH intensity increases home-to-work enrichment.

### 2.4. Boundary conditions of the WFH intensity—positive spillovers relationship

Greenhaus and Powell (2006, p. 86) propose that resources and affect emerging from role A are more likely to “promote high performance in Role B when Role B is highly salient than when it is not highly salient.” Again, we follow boundary theory (Ashforth et al., 2000), which takes the opposite perspective: individuals are highly motivated to enact highly central roles while being engaged in other roles, such that enrichment is most likely to happen from role A to role B if role A is highly central to the individual. For instance, if role A (e.g., family) is highly salient, individuals are motivated to create permeable boundaries around role B (e.g., work), such that role A-related permeations into role B are permitted. For example, one might encourage his or her partner to call anytime while being at work. This “enactment effect” (Capitano et al., 2017; Capitano and Greenhaus, 2018), as posited by boundary theory, has received meta-analytic support (Lapierre et al., 2018): work centrality is more strongly related to work-to-family enrichment (*ρ* = 0.38, 95% CI [0.24, 0.52]) than to family-to-work enrichment (*ρ* = 0.05, 95% CI [−0.18, 0.07]), and family centrality is more strongly related to family-to-work enrichment (*ρ* = 0.21 95% CI [0.07, 0.36]) than to work-to-family enrichment (*ρ* = 0.07 95% CI [−0.03, 0.16]). The longer individuals work from home, the longer both roles are simultaneously accessible and thus increasingly more possibilities exist to enrich one another. For example, employees with a strong work centrality who work from home may be able to benefit longer from the positive work-related emotions because they are motivated to share such positive experiences with their family members (which is an example of work-to-home enrichment). We hypothesize:

*Hypothesis* 6a: Work centrality moderates the positive effect of WFH intensity on work-to-home enrichment, such that the effect is strengthened at high levels of work centrality (relative to low levels).

*Hypothesis* 6b: Home centrality moderates the positive effect of WFH intensity on home-to-work enrichment, such that the effect is strengthened at high levels of home centrality (relative to low levels).

### 2.5. The relevance of inter-role spillovers for satisfaction outcomes in the home and work domain

Operating through opposing spillover paths, WFH intensity can also influence employees’ home- and work-related satisfaction. Relationship satisfaction is an important construct representing the home domain. Romantic relationships were particularly important during the pandemic when individuals could not easily visit friends or relatives and spent (or had to spend) more time with their partners than usual, with both positive and negative consequences. For instance, the German Federal Statistical Office (Statistisches Bundesamt, 2021) reports a slightly reduced divorce rate in 2020 (as compared to 2019), but the Federal Criminal Police Office (Bundeskriminalamt, 2021) registered a sizable increase of cases of domestic violence. Relationship satisfaction is a good indicator of such dynamics, as it is often “the final pathway that leads to relationship breakdown” (Fincham et al., 2018, p. 422). It is also well suited for our purpose because it can be measured independent of one’s broader family and household configuration (existence of children, parents, etc.). Relationship satisfaction, an outcome that is not directly related to organizational performance parameters, further allows us to better understand how far-reaching the influence of work policies can be (Kramer and Kramer, 2021).

Job satisfaction, representing the work domain, is probably the most studied work-related construct of the social sciences (Spector, 1997; Judge et al., 2001) and has important behavioral and organizational consequences, such as extra role behaviors, in-role performance, and withdrawal (e.g., absenteeism and turnover).

How do inter-role spillovers affect relationship satisfaction and job satisfaction? According to the domain specificity model, proposed by Frone et al. (1992), spillover effects originate within the source domain and transpire to the receiving domain, where they materialize. For example, work-to-home conflict (work-to-home enrichment) originates in the work domain (e.g., by work conditions) and manifests in the home domain as decreasing (increasing) relationship satisfaction. An alternative lens, the source attribution model (e.g., Shockley and Singla, 2011), suggests that individuals blame (are grateful to) the source domain, where the spillover effect originates. From this perspective, individuals blame (are grateful to) their job if they experience work-to-home conflict (work-to-home enrichment). Therefore, conflicting (enriching) spillovers can also lead to decreasing (increasing) job satisfaction. We integrate both streams and suggest both positive and negative effects of WFH intensity on relationship satisfaction and job satisfaction *via* enriching and negative spillovers. We approach these four relationships empirically by testing the full mediation model from WFH intensity over spillovers to satisfaction outcomes:

*Hypothesis* 7: WFH intensity influences relationship satisfaction through (a) work-to-home conflict, (b) home-to-work conflict, (c) work-to-home enrichment, and (d) home-to-work enrichment.

*Hypothesis* 8: WFH intensity influences job satisfaction through (a) work-to-home conflict, (b) home-to-work conflict, (c) work-to-home enrichment, and (d) home-to-work enrichment.

## 3. Materials and methods

### 3.1. Sample and procedure

We conducted our study in Munich, Germany. Munich is home to approximately 1.5 million inhabitants (more than 6 million in the metropolitan area), and to the headquarter of seven of the 40 German DAX companies, as well as to a large number of global firms of all sizes.^2^ As a reaction to the pandemic, 10,000 of jobs were moved to home offices in and around Munich, immediately and directly affecting the lives of many employees and their respective households.

We aimed to collect representative data from employees who had a romantic relationship at the time of the survey. To reach the target group, we randomly approached Munich households through a two-step sampling procedure. In the first step, 50 of the 755 Munich voting districts were randomly selected, with the selection probability weighted by the number of inhabitants per district. In the second step, a random geographic point within each selected district was located. Between May 12 and May 15, 2020, research assistants took randomly determined routes from these points, inviting every fifth household to participate in the study by dropping a letter in the mailbox. Within each selected district, 100 letters were distributed, delivering basic information about the study, a unique QR code, and a link to an online survey (T1). Later, on July 20, 2020, we invited participants who had provided an email address to participate in a second wave (T2). In the letter, in reminders, and in the survey itself, we encouraged respondents who lived in a household with family members or cohabitants to share the survey link with these other individuals, specifically with a significant other (if there was one). As an incentive, all participants took part in a raffle for vouchers with a total value of €1,500.

Five thousand households were initially approached, and 800 individuals from 580 households completed at least 50% of the first survey (T1), which translates into a household response rate of 11.6%. We excluded individuals who responded to less than 50% of the T1 survey, one individual who responded to the T1 survey twice, and one individual who provided implausible responses. We further restricted the sample to employed individuals who had a romantic relationship. This left us with a final sample of 545 individuals from 375 households in T1, of which 301 individuals from 229 households responded to the repeat survey at T2 (i.e., the individual attrition rate was 44.7%, and the household attrition rate was 38.9%).

The following descriptive statistics are based on the T1 sample. 53.21% were female. The average age was 44.04 years (SD = 12.03); on average, the participants worked 22.59 (SD = 18.18) hours per week from home, and the average weekly hours worked, including overtime, was 32.09 (SD = 16.20). 24.49% were supervisors/managers; most employees worked in the service industry (57.06%). On average, the participants had 0.43 (SD = 0.83) children under 18. 56.51% of the participants were married; 84.77% lived together with their partner. See Appendix A for further details on the representativeness of our sample and attrition analyses.

### 3.2. The context

**Figure 2:**
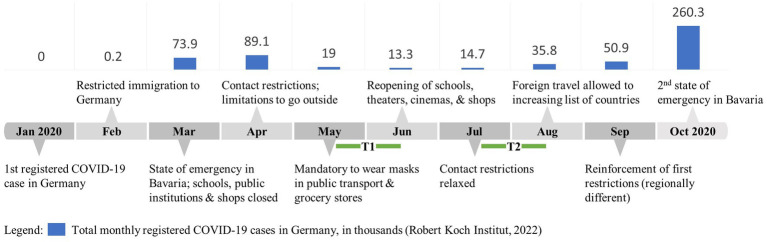
Context of the data collection.

Figure 2 displays the COVID-19 situation in Germany around the data-collection period. The T1 wave of our data was collected about 2 months after the first Bavarian state of emergency. Restrictions were still in place, but the registered COVID-19 cases had already plummeted. The T2 data collection occurred prior to another surge and the second state of emergency. At this time, normality was largely established, contact restrictions were relaxed, and schools, daycare centers and shops were open again. Therefore, our data collections did not occur during extreme situations or periods of volatile adjustments; they rather set in when the work arrangements had already been changed and when individuals had adapted (or were adapting) to them. In this context and at this time, decisions on who would work from home, and when WFH would happen, were made regardless of personal preferences (Chong et al., 2020), thus reducing the self-selection bias inherent in pre-pandemic data and meta-analytic results of WFH.

### 3.3. Measures

#### 3.3.1. Work from home intensity

Following recommendations (Allen et al., 2015), we asked individuals to report the number of weekly WFH hours at both points in time. At T1, we also asked individuals to report their average weekly WFH hours from before the pandemic. See Appendix B for additional descriptive analyses.

#### 3.3.2. Negative spillovers

We measure work-to-home conflict and home-to-work conflict with two four-item German short scales from the German Family Panel (Pairfam Group, 2020). The scales were originally suggested by Carlson et al. (2000) and later translated into German and validated by Wolff and Höge (2011). Example items are: “My work prevents me from doing things with my partner and/or family more than I would like” (work-to-home conflict); and “Conflicts in my personal life reduce my work performance” (home-to-work conflict). Individuals responded to the items on 5-point response scales, ranging from *strongly agree* (1) to *strongly disagree* (5).^3^

#### 3.3.3. Positive spillovers

To measure work-to-home enrichment and home-to-work enrichment, we translated and back-translated (Brislin, 1986) the two three-item scales suggested by Kacmar et al. (2014). The translations were performed with the help of two bilingual German native speakers. Disagreements were resolved *via* discussions between the authors. We replaced “family” from the original scales with “household”/“household member” or “relationship”/“partner,” depending on whether participants indicated that they lived with many others (potentially including their partners) or only with their significant other. Example items are: “The involvement in my household [relationship] puts me in a good mood and this helps me be a better worker” (home-to-work enrichment); and “The involvement in my job makes me feel personally fulfilled and this helps me be a better household member [partner]” (work-to-home enrichment).

#### 3.3.4. Satisfaction outcomes

We measure relationship satisfaction and job satisfaction with the well-accepted single-item measures from the German Socio-Economic Panel study, a prime research resource in the German speaking area (Kantar Public, 2019; “How satisfied are you currently with your relationship?” and “How satisfied are you currently with your job?”). Single-item satisfaction measures often correlate strongly with multi-item measures of the same constructs and can have strong reliability and validity metrics. Research has demonstrated that both single-item measures of relationship satisfaction (Fülöp et al., 2020) and job satisfaction (e.g., Wanous et al., 1997) have adequate psychometric properties, so we are confident in the benefits (low cognitive tax, face validity) of the measures. Both satisfaction measures have ten-point response scales, ranging from *very dissatisfied* (1) to *very satisfied* (10).

#### 3.3.5. Workplace characteristics at home

We measure the two workplace characteristics at home with the following items: “At home, I have optimal equipment [e.g., internet connection, computer] to perform my work tasks” (work equipment); and “At home, I have a room where I am not disturbed when I work” (spatial separation).

#### 3.3.6. Centrality of the work and home domains

We measure work and home centrality using the two two-item scales by Kossek et al. (2012). An example item is “I invest a large part of myself in my work” (work centrality) and “People see me as highly focused on my family/relationship” (home centrality).

### 3.4. Common method bias

To deal with common method variance, we follow the recommendations of Podsakoff et al. (2012). Specifically, we employ different response formats, such as an hour raw count of WFH intensity and varying Likert-type response scales for the spillovers and satisfaction outcomes, which should effectively reduce artifactual inter-construct correlations. In addition, the satisfaction measures were collected at T2, 8 weeks after T1, to create temporal separation.

### 3.5. Analytical strategy

We first use confirmatory factor analysis (CFA) to establish the discriminant validity of the measures. To test hypotheses 1–2 and 4–5, we estimate the direct effects of WFH intensity on inter-role spillovers using structural equation modeling (SEM). To test the conditional effects (hypotheses 3a–3b and 6a–6b), we include the moderators: work equipment and room conditions, and home centrality and work centrality.^4^ To test the influence of spillovers on relationship satisfaction and job satisfaction, we conduct (moderated) multiple mediation analyses. Multiple mediation analysis examines the indirect effects of WFH intensity on satisfaction through inter-role spillovers, and can test whether the spillovers (while controlling for each other) simultaneously mediate the indirect effect (Preacher and Hayes, 2008). All analyses include robust standard errors clustered at the couple level to account for the non-independence of observations where both couple members answered our survey.

Following Graham (2009) and Newman (2003, 2014), missing values in all inferential analyses are handled with a full information maximum likelihood (FIML) procedure. The (moderated) multiple mediation analyses follow the procedures outlined in Preacher and Hayes (2008) and Preacher et al. (2007). Due to the non-normal distribution of the products of coefficients, we calculate confidence intervals for the indirect effects using a parametric resampling (Monte Carlo) approach (MacKinnon et al., 2004) with 20,000 repetitions. All estimations were conducted in the statistical environment *R* (v3.6.1; R Core Team, 2019), using the *lavaan* (v0.6–6; Rosseel, 2012) and *semTools* (v0.5–2; Jorgensen et al., 2020) packages.

### 3.6. Control variables

To alleviate concerns of omitted variable bias and to investigate alternative explanations, we estimate our models both with and without several control variables. We include as controls:

Individual segmentation preferences using the German version of the four-item measure developed by Kreiner (2006) and translated into German by Janke et al. (2014). An example item is: “I do not like work issues creeping into my home life.” Preferences for segmentation (vs. integration) of the work and home domains can cause people to spend less (more) time working from home (Ashforth et al., 2000), and they are probably also related to spillovers (McNall et al., 2015; e.g., Lapierre et al., 2016). Following previous studies of inter-role spillovers and WFH (e.g., Delanoeije et al., 2019), we control for sex (1 = *female*), individual monthly income after taxes (1 = less than € 1,000; 5 = more than €4,000 EUR; at intervals of 1,000 EUR), number of children younger than 18 living at home, age (in years), marital status (1 = *married*), and whether the respondent lived together with his or her significant other (1 = *living together*). We control for the respondents’ household composition (relationship/partner vs. household/household member; 1 = *household*), and for employment characteristics, namely whether the participant was a supervisor (1 = *supervisor*) and whether he or she was full-time or part-time employed (part-time is defined as working less than 35 h per week) (1 = *part-time*). We further include two dummy variables to indicate whether the participant was self-employed (1 = *contract: self-employed*) or had some “other” type of employment (1 = *contract: other*), with *regular employment* as the base category. We use three industry dummies for the *primary and secondary industry* (reference category), *tertiary industry*, and the *public sector*. We also control for pandemic-related task changes: “To what extent did your daily work activities change due to the Corona crisis?” using a 5-point response scale ranging from *my activities did not change at all* (1) to *my activities are completely different now* (5), and include a dummy for individuals on a Government sponsored reduced working hours scheme (German “Kurzarbeit”) (1 = *Kurzarbeit*). Finally, we include the number of hours that individuals had worked from home before the pandemic, capturing the individuals’ familiarity with WFH.^5^

Our results did not qualitatively change when the controls were included. The changes in the standardized coefficients of interest (for example for H1-H2 and H4-H5) were smaller than 0.1, the threshold recommended by Becker (2005). We therefore follow recent advice (Becker, 2005; Carlson and Wu, 2012; Butts et al., 2015) and present our final models without the control variables, but include the models with the full set of control variables in the Appendix (Supplementary Table D.1).

## 4. Results

### 4.1. Confirmatory factor analysis and correlations

The CFA tests whether the four inter-role spillover measures, individual segmentation preferences (a latent control variable), and work centrality and home centrality (two hypothesized moderators) are conceptually different. Because the work-to-home conflict and home-to-work conflict measures have two sub-dimensions each (i.e., stress and time), we modeled the sub-dimensions first and the superordinate work-to-home conflict and home-to-work conflict factors second. The model had a good fit to the data (*χ*^2^[185] = 384.1, comparative fix index [CFI_Robust_] = 0.959, root mean square error of approximation [RMSEA_Robust_] = 0.046, Tucker-Lewis index [TLI_Robust_] = 0.949). Table 1 presents the means, correlations, and standard deviations of all variables, as well as ω (omega) reliabilities for all multi-item constructs. All constructs showed acceptable to good reliabilities (*ω*_Min–Max_ = 0.69–0.89).

**Table 1:** Descriptive statistics and pairwise correlations across study main variables.

	Mean (*SD*)	(1)	(2)	(3)	(4)	(5)	(6)	(7)	(8)	(9)	(10)	(11)
Independent variable
(1) WFH intensity	22.59 (18.18)	–										
Control variables
(2) Segmentation Pref.	3.85 (1.00)	−0.09	(0.89)									
(3) Pre-pandemic WFH	6.21 (11.08)	0.36	−0.18	–								
(4) Female	0.53	−0.12	0.07	0.03	–							
(5) Supervisor	0.24	−0.02	−0.15	0.02	−0.15	–						
(6) Kurzarbeit	0.18	−0.19	0.02	−0.02	0.09	−0.01	–					
(7) No. children < 18	0.43 (0.83)	−0.09	0.02	−0.04	−0.04	0.01	0.04	–				
(8) Income	3.28 (1.23)	0.38	−0.07	0.05	−0.36	0.27	−0.26	−0.01	–			
(9) Age	44.04 (12.03)	−0.11	−0.13	0.08	−0.03	0.16	−0.06	0.04	0.19	–		
(10) Married	0.57	−0.04	−0.08	0.02	−0.06	0.05	0.03	0.39	0.10	0.40	–	
(11) Living together	0.85	0.03	−0.05	0.04	−0.01	0.04	−0.03	0.11	0.11	0.09	0.32	–
(12) Task change	1.98 (1.09)	−0.16	−0.08	−0.04	0.13	0.09	0.20	0.01	−0.15	−0.06	−0.02	0.00
Mediators
(13) WHE	3.28 (1.01)	0.02	−0.29	0.14	0.15	0.09	0.04	0.04	−0.06	0.11	0.11	0.05
(14) HWE	3.60 (0.98)	0.11	−0.05	0.06	0.14	0.01	0.04	−0.08	−0.01	−0.11	−0.02	0.08
(15) WHC	2.50 (0.92)	0.15	0.12	−0.01	−0.02	0.12	−0.06	−0.06	0.10	−0.10	−0.04	0.04
(16) HWC	1.77 (0.79)	−0.02	0.06	0.04	0.01	−0.11	0.06	0.17	−0.17	−0.16	0.04	−0.10
Moderators
(17) Spatial situation	3.56 (1.50)	0.18	−0.16	0.12	−0.07	0.08	−0.06	−0.18	0.16	0.14	0.02	−0.02
(18) Work equipment	4.08 (1.23)	0.16	−0.16	0.13	−0.02	0.05	−0.07	0.00	0.02	0.03	0.05	0.01
(19) Work centrality	3.55 (0.92)	0.12	−0.25	0.13	−0.03	0.22	−0.07	−0.17	0.12	0.07	0.01	0.06
(20) Home centrality	3.52 (0.89)	−0.10	0.07	−0.04	0.12	−0.10	0.13	0.10	−0.23	−0.14	0.05	0.06
Dependent variables
(21) Relationship Sat. _T2_	8.22 (2.23)	0.04	−0.02	0.06	−0.02	0.04	−0.14	−0.12	0.07	−0.03	−0.04	0.08
(22) Job Sat. _T2_	6.83 (2.45)	0.01	−0.20	−0.07	0.01	0.00	−0.11	0.08	0.06	0.09	0.17	0.08

	(12)	(13)	(14)	(15)	(16)	(17)	(18)	(19)	(20)	(21)	(22)
Mediators
(13) WHE	0.08	(0.84)									
(14) HWE	0.03	0.43	(0.83)								
(15) WHC	0.16	−0.20	−0.08	(0.73)							
(16) HWC	0.06	−0.04	−0.15	0.19	(0.84)						
Moderators
(17) Spatial situation	−0.06	0.14	0.08	−0.03	−0.11	–					
(18) Work equipment	−0.09	0.15	0.10	−0.03	0.02	0.43	–				
(19) Work centrality	0.04	0.16	0.09	0.30	−0.09	0.11	0.11	(0.69)			
(20) Home centrality	0.04	0.09	0.15	−0.11	0.10	−0.06	−0.03	−0.06	(0.67)		
Dependent variables
(21) Relationship Sat. _T2_	−0.10	0.09	0.23	−0.18	−0.20	0.19	0.04	−0.10	0.14	–	
(22) Job Sat. _T2_	−0.04	0.37	0.17	−0.26	−0.07	0.07	0.03	−0.07	0.07	0.27	–

N_T1_ = 545, N_T2_ = 301. In the dataset collected at T1, correlations equal or above *r* = |0.07| are significant at *p* < 0.10, equal or above *r* = |0.09| at *p* < 0.05, equal or above *r* = |0.12| at *p* < 0.01, and equal or above *r* = |0.15| at *p* < 0.001 levels. In the dataset including outcome variables measured at T2, the following values apply (same order): *r* = |0.10|, *r* = |0.12|, *r* = |0.17|, *r* = |0.19|. *ω* reliabilities are in the diagonal (multi-item constructs). To simplify the table, industry (2 dummies), contract-type dummies (part-time dummy, and two dummies indicating self-employment and “other” with employed as base category), and a dummy indicating the point of reference of the WHE and HWE items (household composition) are not included but used as controls in all analyses.

### 4.2. Results of hypothesis tests

Hypotheses 1–2 and 4–5 suggest that WFH intensity is positively associated with work-to-home conflict, home-to-work conflict, work-to-home enrichment, and home-to-work enrichment. In line with Hypotheses 1 and 5, we find a significant and positive effect of WFH intensity on work-to-home conflict (*b* = *0*.008, *p* < 0.01) and home-to-work enrichment (*b* = *0*.005, *p* < 0.05). Hypotheses 2 and 4 do not receive support, as both coefficients are not significant at conventional levels (Table 2).

**Table 2:** Direct unconditional and conditional effects of WFH intensity on work-home spillovers.

Variable	WHC		HWC		WHE		HWE	
	H1	H3a	H2	H3b	H4	H6a	H5	H6b
WFH intensity	0.008^**^	0.009^**^	–0.000	0.000	0.001	0.000	0.005^*^	0.006^**^
Work equipment		–0.062						
WFH intensity × work equipment		–0.005^*^						
Room conditions				–0.051^*^				
WFH intensity × room conditions				0.001				
Work centrality						0.188^***^		
WFH intensity × work centrality						0.000		
Home centrality								0.158^***^
WFH intensity × home centrality								0.006^**^

*N* = 545. ^*^p < 0.05, ^**^p < 0.01, ^***^p < 0.001. Unstandardized coefficients. Robust standard errors clustered at the couple level. WFH intensity and all moderators mean-centered. Models including the full set of control variables are in the Appendix (Supplementary Table D.1). WHC = work-to-home conflict, HWE = home-to-work enrichment, HWC and WHE accordingly.

**Figure 3:**
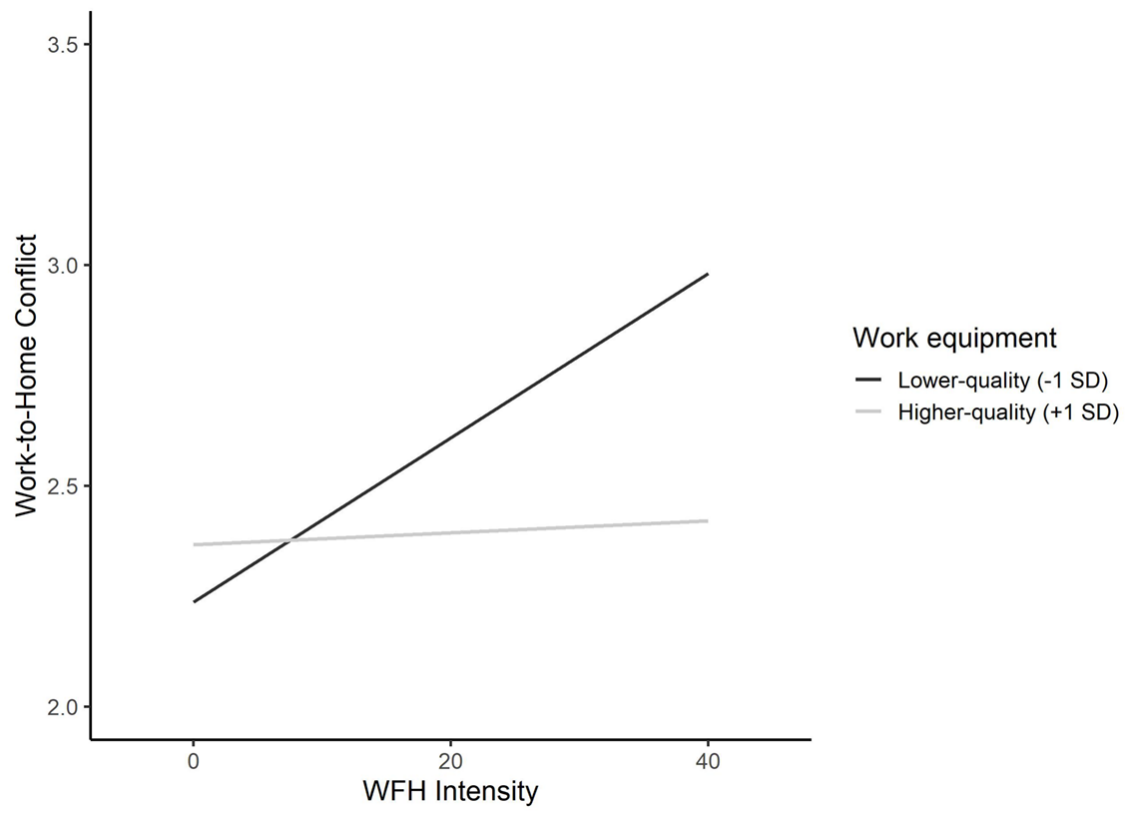
Effects of WFH intensity on work-to-home conflict at lower- and higher-quality levels of work equipment.

Next, we tested Hypotheses 3a and 3b, predicting that the work equipment (3a) and room conditions (3b) moderate the positive effect of WFH intensity on work-to-home conflict and home-to-work conflict, respectively. All continuous variables were mean-centered to avoid bias due to nonessential collinearity between the predictors and the multiplied terms (Dalal and Zickar, 2012). In support of H3a, we find a significant and negative moderation effect of the quality of one’s work equipment on work-to-home conflict (*b* = −0.005, *p* < 0.05; Figure 3 depicts the simple slopes). Specifically, we find that a 1 h-increase of WFH increases the employees’ work-to-home conflict by 0.017 units (95% CI [0.010, 0.025]) if they have low-quality work equipment (-1SD), whereas the effect is not statistically significant for employees with high-quality work equipment (+1SD) (*b* = *0*.002, 95% CI [−0.005, 0.009]). We do not find support for Hypothesis 3b, though.

**Figure 4:**
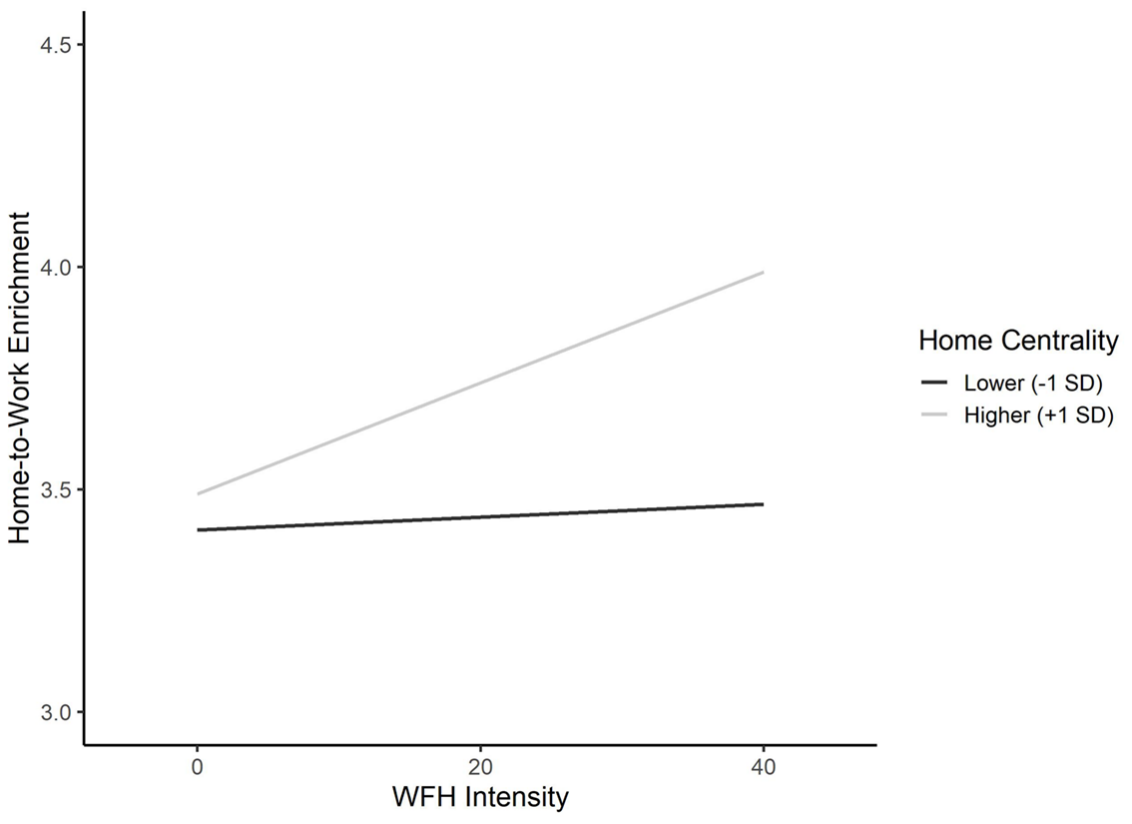
Effects of WFH intensity on home-to-work enrichment at lower- and higher-levels of home centrality.

Hypotheses 6a and 6b proposed that work centrality and home centrality moderate the positive effect of WFH intensity on work-to-home enrichment and home-to-work enrichment. We do not find a significant moderation of the WFH intensity—work-to-home enrichment relationship and therefore reject H6a. In line with hypothesis 6b, we find that home centrality moderates the positive effect of WFH intensity on work-to-home enrichment (*b* = *0*.006, *p* < 0.01). Specifically, we find that employees, who report their home to be less central (–1SD) experience little increase in home-to-work enrichment when WFH-hours increase (*b* = *0*.002, 95% CI [−0.005, 0.009]). Employees who report their home to be more central (+1SD), however, can benefit from WFH. On average, their home-to-work enrichment increases by 0.012 units with each additional hour worked from home (95% CI [0.005, 0.019]). Figure 4 plots the effects.

**Figure 5:**
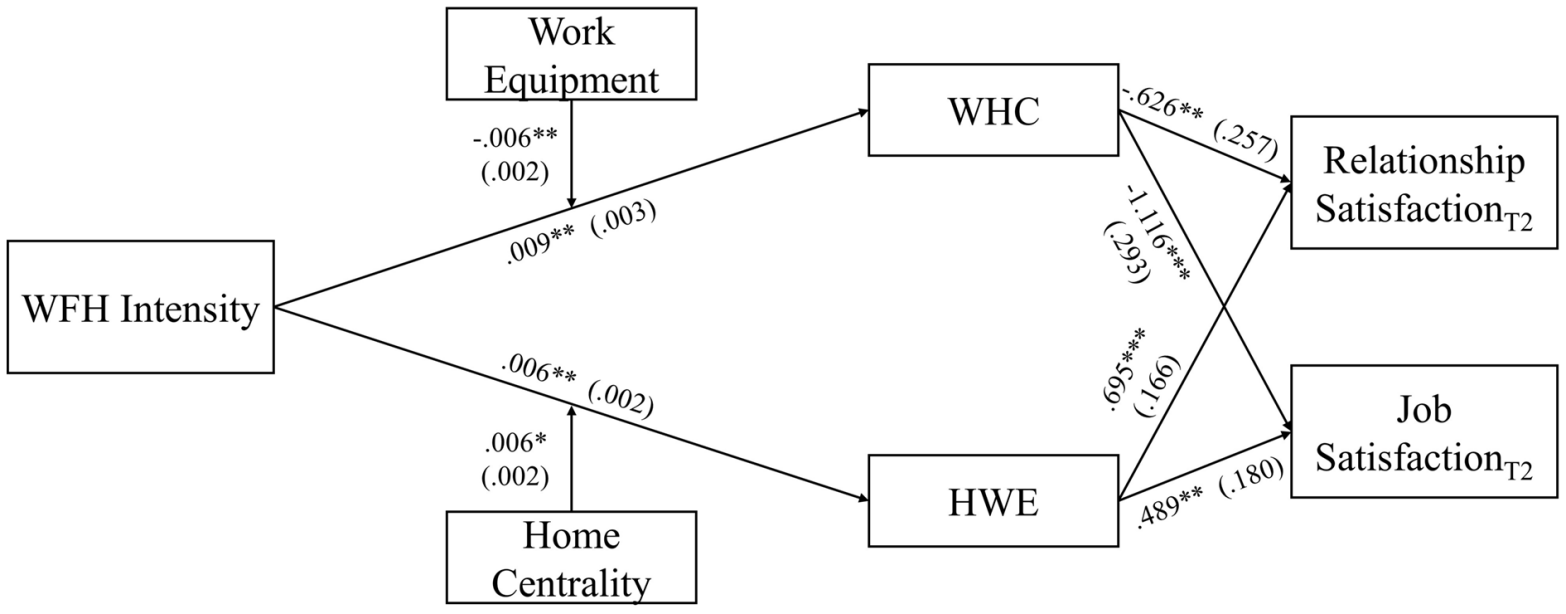
Effects of WFH intensity on relationship and job satisfaction *via* work-to-home conflict and home-to-work enrichment in a moderated multiple mediation model.

The analyses reveal that WFH intensity has adverse *and* beneficial effects. The positive effect of WFH intensity on work-to-home conflict implies a negative effect of WFH intensity on relationship and job satisfaction, whereas the positive effect on home-to-work enrichment implies a positive effect on both satisfaction outcomes. We use multiple mediation models (Preacher and Hayes, 2008) to examine whether these significant paths exist above and beyond each other. We do not include home-to-work conflict and work-to-home enrichment, because we did not find direct effects of WFH intensity on these inter-role spillovers.^6^ Specifically, we reject hypotheses 7b, 7c, 8b and 8c. Due to non-significant a-paths, the indirect effects of WFH intensity on relationship satisfaction and job satisfaction *via* work-to-home conflict or home-to-work enrichment are statistically non-significant. To test hypotheses 7a, 7d, 8a, and 8d, we estimated a multiple mediation model in which we included relationship satisfaction and job satisfaction as outcomes, and the significant mediation paths from our main analyses, i.e., work-to-home conflict and home-to-work enrichment. Figure 5 presents the estimated paths, and Table 3 reports the indirect effects of the multiple mediation analysis.

**Table 3:** Indirect effects of WFH intensity on satisfaction outcomes measured after 8 weeks in multiple mediation models.

	Relationship satisfaction _T2_			Job satisfaction _T2_		
		95% CI			95% CI	
Mechanism	Indirect effect	Lower	Upper	Indirect effect	Lower	Upper
Multiple mediation
WHC	−0.005	−0.011	−0.001	−0.009	−0.016	−0.003
HWE	0.004	0.0004	0.007	0.003	0.0001	0.006

N_T1_ = 545, N_T2_ = 301. Unstandardized indirect effects and Monte Carlo (20,000) CIs are reported. CI = confidence interval; WHC = work-to-home conflict, HWE = home-to-work enrichment.

We find that work-to-home conflict mediates the negative effect of WFH intensity on relationship satisfaction (*IND* = −0.005, 95% CI [−0.011, −0.001]) and job satisfaction (*IND* = *−0*.009, 95% CI [−0.016, −0.003]). An indirect effect tells us “how much two cases that differ by a unit on X are estimated to differ on Y as a result of X’s influence on M, which in turn influences Y” (Hayes, 2017, 95). For example, if two employees have zero versus 22.59 WFH hours per week (the sample mean), our model predicts that they will differ by approximately 0.20 job satisfaction units, based only on the mediation through work-to-home conflict and neglecting other potential pathways. This represents about a tenth of a standard deviation of job satisfaction, a small but noteworthy effect. The indirect effects of WFH intensity *via* home-to-work enrichment on relationship satisfaction (*IND* = *0*.004, 95% CI [0.0004, 0.007]) and job satisfaction (*IND* = *0*.003, 95% CI [0.0001, 0.006]) are positive and statistically significant.

Including the previously statistically significant moderators work equipment and home centrality into a moderated multiple mediation model confirms the findings; individuals can alleviate the adverse effects of WFH intensity on satisfaction outcomes through work-to-home conflict by higher-quality work equipment; likewise, individuals who regard their home as more central have weaker negative effects and can even enhance positive effects through increased home-to-work enrichment (see Figure 5).

## 5. Discussion

Building on boundary theory (Ashforth et al., 2000), this paper argues that WFH intensity results in negative and positive spillovers, i.e., work-to-home conflict, home-to-work conflict, work-to-home enrichment, and home-to-work enrichment. For each effect, we propose a boundary condition. We hypothesize that workspace characteristics attenuate the relationship between WFH intensity and work-to-home conflict and home-to-work conflict. In addition, we propose that higher home centrality and work centrality enhance the positive relationship between WFH intensity and home-to-work enrichment and work-to-home enrichment. We test the relevance of these effects for satisfaction outcomes, namely relationship satisfaction and job satisfaction.

Using a two-step random sampling procedure in Munich, Germany, we obtained a diverse sample of 545 employees which we surveyed twice. In line with our hypotheses, we find a significant positive effect of WFH intensity on work-to-home conflict (*H1*) and home-to-work enrichment (*H5*). Multiple mediation analyses reveal that these effects exist simultaneously and have negative (*via* work-to-home conflict, *H7a, H8a*) and positive (*via* home-to-work enrichment, *H7d, H8d*) implications for employees’ relationship satisfaction and job satisfaction. Surprisingly, we do not detect a significant effect of WFH intensity on home-to-work conflict (*H2*) and work-to-home enrichment (*H4*). We further find that the positive effects of WFH intensity on work-to-home conflict and home-to-work enrichment can be attenuated through high-quality work equipment (*H3a*) and are stronger for individuals with a high home centrality (*H6b*), respectively.

We contribute to several current discussions in the literature. First, our finding of opposing effects of WFH intensity on the work-home interface confirms that WFH is a work stressor *and* a work-related resource (Huyghebaert-Zouaghi et al., 2022). This aligns with boundary theory (Ashforth et al., 2000), which predicts positive and negative spillovers as a consequence of role integration. The heterogeneous consequences of WFH intensity warn against a one-sided focus on WFH.

Second, we contribute to the ongoing debate about positive spillovers by analyzing the role of domain centrality as a moderator candidate. Following Greenhaus and Powell (2006) home-to-*work* enrichment would be more likely to result from WFH intensity when the *work* domain is central. Enactment-effect logic (Capitano et al., 2017; Capitano and Greenhaus, 2018) takes a different perspective, according to which individuals seek to enact highly salient roles in other roles. Therefore, individuals with high *home* centrality should experience higher *home*-to-work enrichment when the WFH intensity increases. Our findings support the latter line of reasoning, and are in line with recent meta-analytic results (Lapierre et al., 2018).

Third, we fill a gap and study how WFH intensity relates to relationship satisfaction. Research has mostly neglected the non-work outcomes of work arrangements, taking an organizational perspective (Kramer and Kramer, 2021). However, spillovers from the work to the home domain are increasingly determining how employees experience their jobs and select their employers, making it clear that seemingly non-work outcomes have both individual-level *and* organizational-level relevance. Given the negative views on WFH as portrayed in the media (e.g., Dunn, 2020), it is important to understand whether and how WFH affects the home domain.

Fourth, we add to current research that examines whether the pre-pandemic WFH literature can be generalized to the new, post-pandemic era of WFH (Kniffin et al., 2021). In line with other pandemic-related research (Chong et al., 2020; Leroy et al., 2021), we find a robust and significant positive relationship between WFH and work-to-home conflict, leading to a negative indirect effect of WFH on job satisfaction, whereas pre-pandemic WFH research has found an opposite effect (e.g., Gajendran and Harrison, 2007; Allen et al., 2013). A key difference between pre- and post-pandemic WFH research seems to be that current WFH arrangements do not always increase employees’ flexibility (probably due to lower self-selection autonomy); rather, they seem to decrease the employees’ boundary control (consistent with Lapierre et al., 2016). Surprisingly, we failed to find a significant effect of WFH on home-to-work conflict, which contradicts much of the public debate (e.g., Dunn, 2020) but aligns with the pre-pandemic literature (Allen et al., 2013).

### 5.1. Practical implications

As companies plan to reduce office space and fixed costs, WFH is here to stay (Aksoy et al., 2023). Practitioners argue that more WFH intensity is not exclusively beneficial for companies, but has also positive implications for employees’ work and private life (e.g., Bundeskriminalamt, 2021). Indeed, meta-analyses have found small but positive effects of WFH intensity on the work-home interface and job satisfaction before the pandemic (Gajendran and Harrison, 2007; Allen et al., 2015). Our findings suggest that this may be true for some employees; more importantly, however they suggest that reality is more complex. On the one hand, higher WFH intensity increases employees’ home-to-work enrichment, which correlates positively with job satisfaction and relationship satisfaction. However, increased WFH intensity also triggers work-to-home conflict. Since work-to-home conflict impairs central work and family outcomes, it is necessary (for employers and employees!) to reconsider for whom the positive aspects outweigh the negative aspects, and how to manage employees who work from home. In this challenging situation, our study suggests some promising interventions.

Our findings indicate that employees’ specific situations matter. Employees who have high family centrality are more likely to reap the benefits of WFH. To minimize the harmful effect of WFH intensity on the work-home interface, organizations can provide employees with improved work equipment. We encourage companies and policymakers to study further key mechanisms that could crowd out the negative and crowd in the positive consequences of WFH. Some companies have already taken steps in this direction by designing software and IT-tools that enable individuals to better manage their work-home boundaries (e.g., Liu, 2020), or by providing employees with a budget for office tools and equipment so that they have what the need to continue working from home (e.g., Kelly, 2021).

### 5.2. Limitations

Our study has limitations. First, our model is static, with predictors and mediators measured at one point in time, and outcomes measured 8 weeks later at another point in time. This data structure is not sufficient to adopt a dynamic view of inter-role spillovers, a call we echo (Allen et al., 2019).

Second, we conducted our study at the beginning of the COVID-19 pandemic. Although this setting helped us to control for some empirical issues (e.g., self-selection, reverse causality; e.g., Maxwell and Cole, 2007), we cannot rule out all potential biases. For example, omitted variables may bias the results. For example, employees’ resilience and coping skills may affect both WFH intensity during the pandemic (assuming that employees have some autonomy to determine their WFH hours, which is not clear, however) and negative spillovers. Reverse causality can also be an issue. For example, individuals suffering from high work-to-home conflict might be less inclined to choose WFH (again assuming that employees have a choice). A quasi-experimental design, with initial measures taken before the pandemic and follow-up measures taken during/after the pandemic, would have been preferred.

Third, the contingency view we developed focuses on employee-based moderators, but neglects organization-based and occupation-based contingencies. We echo calls to examine how organizational and occupational characteristics influence the experience of WFH (Kossek and Lautsch, 2018).

Finally, generalizability may be an issue. Our study was conducted during the first months of the pandemic in Munich, Germany. The timing of the study allowed us to test the effect of WFH at its peak, but the longitudinal stability of the effects remains unclear. In addition, the inhabitants of Munich can be described by the WEIRD acronym (i.e., they represent a Western, Educated, Industrialized, Rich, and Democratic society; Henrich et al., 2010). More studies are needed to obtain a broader, cross-cultural picture of the role integration demands that the pandemic has triggered.

## 6. Conclusion

Collecting data from a diverse sample of 545 working employees who were in a romantic relationship, we found that WFH intensity impacts individuals’ work-home interface, opening the door for negative and positive spillovers and ultimately influencing employees’ satisfaction with their home and work domains. We find that individuals with high a high home centrality experience higher home-to-work enrichment with increasing WFH, thereby providing sound empirical evidence for the enactment-effect (Capitano et al., 2017; Capitano and Greenhaus, 2018). We further found that the negative effects of WFH intensity through work-to-home conflict could be mitigated through higher-quality work equipment, an actionable insight for both managers and WFH employees.

In summary, these findings propose answers to our initially presented research questions: The consequences of WFH are multifaceted with beneficial and adverse effects on employees’ work-home interface. In addition, employees’ experiences with WFH depend on boundary conditions such as the work environment and personal differences.

## Data availability statement

The datasets presented in this article are not readily available because the raw data supporting the conclusions of this article will be made available by the authors upon request. Requests to access the datasets should be directed to boelingen@lmu.de.

## Ethics statement

Ethical review and approval was not required for the study on human participants in accordance with the local legislation and institutional requirements. The patients/participants provided their written informed consent to participate in this study.

## Author contributions

All authors listed have made a substantial, direct, and intellectual contribution to the work and approved it for publication.

## Conflict of interest

The authors declare that the research was conducted in the absence of any commercial or financial relationships that could be construed as a potential conflict of interest.

## Publisher’s note

All claims expressed in this article are solely those of the authors and do not necessarily represent those of their affiliated organizations, or those of the publisher, the editors and the reviewers. Any product that may be evaluated in this article, or claim that may be made by its manufacturer, is not guaranteed or endorsed by the publisher.

## Footnotes

^1^We use the term “work from home” for reasons of readability and consistency but acknowledge and include in our review the general literature on telework and telecommuting, which can be performed in other places than at home (for a review of definitions see Allen et al., 2015).

^2^For instance, Munich is also home to the headquarters of 10 of 50 MDAX firms, 8 of 30 TecDAX firms, and 11 of 50 SDAX firms, highlighting the economic power of the city and the metropolitan region more generally.

^3^Unless indicated otherwise, all item measures have the same response scale.

^4^We predict a single boundary condition for each main effect. However, our proposed boundary conditions might moderate the relationships between WFH intensity and other inter-role spillovers as well. We test a complete model (with all possible moderations) and report the results in the Appendix E.

^5^When this control variable is included, the WFH intensity effect becomes a change in WFH intensity effect (on all outcomes).

^6^We also do not find indirect effects of WFH intensity on relationship satisfaction and job satisfaction *via* home-to-work conflict (*H7b, H8b*) and work-to-home enrichment (*H7c, H8c*) in mediation analyses. The insignificant effects of WFH intensity on home-to-work conflict and work-to-home enrichment are not moderated by the spatial situation (H3b) or work centrality (H6a).

## References

[ref1] Aksoy C. G. Barrero J. M. Bloom N. Davis S. J. Dolls M. Zarate P. (2023). Working from home around the world. Cent. Econ. Perform. Discuss. Pap. 1920

[ref2] Allen T. D. Cho E. Meier L. L. (2014). Work–family boundary dynamics. Annu. Rev. Organ. Psychol. Organ. Behav. 1, 99–121. doi: 10.1146/annurev-orgpsych-031413-091330

[ref3] Allen T. D. French K. A. Braun M. T. Fletcher K. (2019). The passage of time in work-family research: toward a more dynamic perspective. J. Vocat. Behav. 110, 245–257. doi: 10.1016/j.jvb.2018.11.013

[ref4] Allen T. D. Golden T. D. Shockley K. M. (2015). How effective is telecommuting? Assessing the status of our scientific findings. Psychol. Sci. Public Interest 16, 40–68. doi: 10.1177/1529100615593273, PMID: 26403188

[ref5] Allen T. D. Johnson R. C. Kiburz K. M. Shockley K. M. (2013). Work-family conflict and flexible work arrangements: deconstructing flexibility. Pers. Psychol. 66, 345–376. doi: 10.1111/peps.12012

[ref6] Allen T. D. Merlo K. Lawrence R. C. Slutsky J. Gray C. E. (2021). Boundary management and work-nonwork balance while working from home. Appl. Psychol. 70, 60–84. doi: 10.1111/apps.12300

[ref7] Ashforth B. E. Kreiner G. E. Fugate M. (2000). All in a day’s work: boundaries and micro role transitions. Acad. Manag. Rev. 25, 472–491. doi: 10.5465/amr.2000.3363315

[ref8] Becker T. E. (2005). Potential problems in the statistical control of variables in organizational research: a qualitative analysis with recommendations. Organ. Res. Methods 8, 274–289. doi: 10.1177/1094428105278021

[ref9] Bennett A. A. Gabriel A. S. Calderwood C. (2020). Examining the interplay of micro-break durations and activities for employee recovery: a mixed-methods investigation. J. Occup. Health Psychol. 25, 126–142. doi: 10.1037/ocp0000168, PMID: 31464460

[ref10] Bosch C. Sonnentag S. Pinck A. S. (2018). What makes for a good break? A diary study on recovery experiences during lunch break. J. Occup. Organ. Psychol. 91, 134–157. doi: 10.1111/joop.12195

[ref11] Brislin R. W. (1986). “The wording and translation of research instruments” in Field Methods in Cross-cultural Research Cross-cultural Research and Methodology Series. eds. Lonner W. J. Berry J. W. (Thousand Oaks, CA: Sage Publications, Inc), 137–164.

[ref12] Bundeskriminalamt (2021). Partnerschaftsgewalt, Kriminalstatistische Auswertung-Berichtsjahr 2020. Wiesbaden.

[ref13] Butts M. M. Becker W. J. Boswell W. R. (2015). Hot buttons and time sinks: the effects of electronic communication during nonwork time on emotions and work-nonwork conflict. Acad. Manag. J. 58, 763–788. doi: 10.5465/amj.2014.0170

[ref14] Capitano J. DiRenzo M. S. Aten K. J. Greenhaus J. H. (2017). Role identity salience and boundary permeability preferences: an examination of enactment and protection effects. J. Vocat. Behav. 102, 99–111. doi: 10.1016/j.jvb.2017.07.001

[ref15] Capitano J. Greenhaus J. H. (2018). When work enters the home: antecedents of role boundary permeability behavior. J. Vocat. Behav. 109, 87–100. doi: 10.1016/j.jvb.2018.10.002

[ref16] Carlson D. S. Kacmar K. M. Williams L. J. (2000). Construction and initial validation of a multidimensional measure of work-family conflict. J. Vocat. Behav. 56, 249–276. doi: 10.1006/jvbe.1999.1713

[ref17] Carlson K. D. Wu J. (2012). The illusion of statistical control: control variable practice in management research. Organ. Res. Methods 15, 413–435. doi: 10.1177/10944281114288

[ref18] Chong S. Huang Y. Chang C.-H. (Daisy) (2020). Supporting interdependent telework employees: a moderated-mediation model linking daily COVID-19 task setbacks to next-day work withdrawal. J. Appl. Psychol. 105, 1408–1422. doi: 10.1037/apl0000843 33271029

[ref19] Clark S. C. (2000). Work/family border theory: a new theory of work/family balance. Hum. Relat. 53, 747–770. doi: 10.1177/0018726700536001

[ref20] Dalal D. K. Zickar M. J. (2012). Some common myths about centering predictor variables in moderated multiple regression and polynomial regression. Organ. Res. Methods 15, 339–362. doi: 10.1177/1094428111430540

[ref21] Delanoeije J. Verbruggen M. Germeys L. (2019). Boundary role transitions: a day-to-day approach to explain the effects of home-based telework on work-to-home conflict and home-to-work conflict. Hum. Relat. 72, 1843–1868. doi: 10.1177/0018726718823071

[ref22] Dunn J. (2020). How to Work From Home Alongside Your Partner Without Losing it. New York, NY. Times.

[ref23] Feeney B. C. Collins N. L. (2001). Predictors of caregiving in adult intimate relationships: an attachment theoretical perspective. J. Pers. Soc. Psychol. 80, 972–994. doi: 10.1037/0022-3514.80.6.972, PMID: 11414378

[ref24] Ferguson M. Carlson D. S. Boswell W. Whitten D. Butts M. M. Kacmar K. M. (2016). Tethered to work: a family systems approach linking mobile device use to turnover intentions. J. Appl. Psychol. 101, 520–534. doi: 10.1037/apl0000075, PMID: 26653530

[ref25] Fincham F. D. Rogge R. Beach S. R. H. (2018). “Relationship satisfaction” in The Cambridge Handbook of Personal Relationships. eds. Vangelisti A. L. Perlman D. (Cambridge, UK: Cambridge University Press), 422–436.

[ref26] Fonner K. L. Stache L. C. (2012). All in a day’s work, at home: teleworkers’ management of micro role transitions and the work–home boundary. New Technol. Work Employ. 27, 242–257. doi: 10.1111/j.1468-005x.2012.00290.x

[ref27] Frone M. R. (2003). “Work-family balance” in Handbook of Occupational Health Psychology. eds. Quick J. C. Tetrick L. E. (Washington, DC: American Psychological Association), 143–162.

[ref28] Frone M. R. Russell M. Cooper M. L. (1992). Prevalence of work-family conflict: are work and family boundaries asymmetrically permeable? J. Organ. Behav. 13, 723–729. doi: 10.1002/job.4030130708

[ref29] Fülöp F. Bőthe B. Gál É. Cachia J. Y. A. Demetrovics Z. Orosz G. (2020). A two-study validation of a single-item measure of relationship satisfaction: RAS-1. Curr. Psychol. 41, 2109–2121. doi: 10.1007/s12144-020-00727-y

[ref30] Gajendran R. S. Harrison D. A. (2007). The good, the bad, and the unknown about telecommuting: Meta-analysis of psychological mediators and individual consequences. J. Appl. Psychol. 92, 1524–1541. doi: 10.1037/0021-9010.92.6.1524, PMID: 18020794

[ref31] Gohmann J. (2020). Eleven tips for working from home alongside your partner during the global pandemic. *New Yorker*. Available at: https://www.newyorker.com/humor/daily-shouts/eleven-tips-for-working-from-home-alongside-your-partner-during-the-global-pandemic. Accessed December 9, 2020.

[ref32] Graham J. W. (2009). Missing data analysis: making it work in the real world. Annu. Rev. Psychol. 60, 549–576. doi: 10.1146/annurev.psych.58.110405.085530, PMID: 18652544

[ref33] Greenhaus J. H. Powell G. N. (2006). When work and family are allies: a theory of work-family enrichment. Acad. Manag. Rev. 31, 72–92. doi: 10.5465/amr.2006.19379625

[ref35] Hayes A. F. (2017). Introduction to Mediation, Moderation, and Conditional Process Analysis: A Regression-based Approach. New York City, NY: Guilford Publications.

[ref36] Henrich J. Heine S. J. Norenzayan A. (2010). Beyond WEIRD: towards a broad-based behavioral science. Behav. Brain Sci. 33, 111–135. doi: 10.1017/S0140525X10000725

[ref37] Huyghebaert-Zouaghi T. Morin A. J. S. Fernet C. Austin S. Gillet N. (2022). Longitudinal profiles of work-family interface: their individual and organizational predictors, personal and work outcomes, and implications for onsite and remote workers. J. Vocat. Behav. 134:103695. doi: 10.1016/j.jvb.2022.103695

[ref38] Ilies R. Wilson K. S. Wagner D. T. (2009). The spillover of daily job satisfaction onto employees’ family lives: the facilitating role of work-family integration. Acad. Manag. J. 52, 87–102. doi: 10.5465/amj.2009.36461938

[ref39] Janke I. Stamov-Roßnagel C. Scheibe S. (2014). Verschwimmen die Grenzen? Auswirkungen von Vertrauensarbeitszeit auf die Schnittstelle von Arbeit und Privatleben. Z. Für Arbeitswissenschaft 68, 97–104. doi: 10.1007/BF03374430

[ref40] Jorgensen T. D. Pornprasertmanit S. Schoemann A. M. Rosseel Y. Miller P. Quick C.. (2020). semTools: Useful tools for structural equation modeling. Available at https://CRAN.R-project.org/

[ref41] Judge T. A. Ilies R. (2004). Affect and job satisfaction: a study of their relationship at work and at home. J. Appl. Psychol. 89, 661–673. doi: 10.1037/0021-9010.89.4.661 15327352

[ref42] Judge T. A. Thoresen C. J. Bono J. E. Patton G. K. (2001). The job satisfaction-job performance relationship: a qualitative and quantitative review. Psychol. Bull. 127, 376–407. doi: 10.1037/0033-2909.127.3.376, PMID: 11393302

[ref43] Kacmar K. M. Crawford W. S. Carlson D. S. Ferguson M. Whitten D. (2014). A short and valid measure of work-family enrichment. J. Occup. Health Psychol. 19, 32–45. doi: 10.1037/a0035123, PMID: 24447219

[ref44] Kantar Public (2019). SOEP-Core–2018: Personenfragebogen, Stichproben A-L3 + N. SOEP Surv. Pap:608.

[ref45] Kelly J. (2021). Salesforce says ‘9-to-5 workday is dead’ and employees will only come into the office one to three days a week. Forbes. Available at: https://www.forbes.com/sites/jackkelly/2021/02/10/salesforce-says-9-to-5-workday-is-dead-and-employees-will-only-come-into-the-office-one-to-three-days-a-week/ (Accessed June 22, 2023).

[ref46] Kniffin K. M. Narayanan J. van Vugt M. (2021). COVID-19 is a moderating variable with its own moderating factors. Ind. Organ. Psychol. 14, 149–151. doi: 10.1017/iop.2021.38

[ref47] Kossek E. E. Dumas T. L. Piszczek M. M. Allen T. D. (2021). Pushing the boundaries: a qualitative study of how stem women adapted to disrupted work–nonwork boundaries during the COVID-19 pandemic. J. Appl. Psychol. 106, 1615–1629. doi: 10.1037/apl0000982, PMID: 34871022

[ref48] Kossek E. E. Lautsch B. A. (2018). Work–life flexibility for whom? Occupational status and work–life inequality in upper, middle, and lower level jobs. Acad. Manag. Ann. 12, 5–36. doi: 10.5465/annals.2016.0059

[ref49] Kossek E. E. Ruderman M. N. Braddy P. W. Hannum K. M. (2012). Work–nonwork boundary management profiles: a person-centered approach. J. Vocat. Behav. 81, 112–128. doi: 10.1016/j.jvb.2012.04.003

[ref50] Kramer A. Kramer K. Z. (2021). Putting the family back into work and family research. J. Vocat. Behav. 126:103564. doi: 10.1016/j.jvb.2021.103564

[ref51] Kreiner G. E. (2006). Consequences of work-home segmentation or integration: a person-environment fit perspective. J. Organ. Behav. 27, 485–507. doi: 10.1002/job.386

[ref52] Kreiner G. E. Hollensbe E. C. Sheep M. L. (2009). Balancing borders and bridges: negotiating the work-home interface via boundary work tactics. Acad. Manag. J. 52, 704–730. doi: 10.5465/amj.2009.43669916

[ref53] Lapierre L. M. Li Y. Kwan H. K. Greenhaus J. H. DiRenzo M. S. Shao P. (2018). A meta-analysis of the antecedents of work-family enrichment. J. Organ. Behav. 39, 385–401. doi: 10.1002/job.2234

[ref54] Lapierre L. M. van Steenbergen E. F. Peeters M. C. W. Kluwer E. S. (2016). Juggling work and family responsibilities when involuntarily working more from home: a multiwave study of financial sales professionals. J. Organ. Behav. 37, 804–822. doi: 10.1002/job.2075

[ref55] Leroy S. Schmidt A. M. Madjar N. (2021). Working from home during COVID-19: a study of the interruption landscape. J. Appl. Psychol. 106, 1448–1465. doi: 10.1037/apl0000972, PMID: 34855421

[ref56] Liu J. (2020). How companies are preparing employees for long-term work-from-home. *CNBC*. Available at: https://www.cnbc.com/2020/08/25/how-companies-are-supporting-work-from-home-until-2021or-forever.html. Accessed October 23, 2020.

[ref57] MacKinnon D. P. Lockwood C. M. Williams J. (2004). Confidence limits for the indirect effect: distribution of the product and resampling methods. Multivar. Behav. Res. 39, 99–128. doi: 10.1207/s15327906mbr3901_4, PMID: 20157642 PMC2821115

[ref58] Massar S. A. A. Ong J. L. Lau T. Ng B. K. L. Chan L. F. Koek D.. (2023). Working-from-home persistently influences sleep and physical activity 2 years after the Covid-19 pandemic onset: a longitudinal sleep tracker and electronic diary-based study. Front. Psychol. 14:1145893. doi: 10.3389/fpsyg.2023.1145893, PMID: 37213365 PMC10196619

[ref59] Maxwell S. E. Cole D. A. (2007). Bias in cross-sectional analyses of longitudinal mediation. Psychol. Methods 12, 23–44. doi: 10.1037/1082-989X.12.1.23 17402810

[ref60] McNall L. A. Masuda A. D. Nicklin J. M. (2009). Flexible work arrangements, job satisfaction, and turnover intentions: the mediating role of work-to-family enrichment. J. Psychol. 144, 61–81. doi: 10.1080/00223980903356073, PMID: 20092070

[ref61] McNall L. A. Scott L. D. Nicklin J. M. (2015). Do positive affectivity and boundary preferences matter for work-family enrichment? A study of human service workers. J. Occup. Health Psychol. 20, 93–104. doi: 10.1037/a0038165, PMID: 25347683

[ref62] Newman D. A. (2003). Longitudinal modeling with randomly and systematically missing data: a simulation of ad hoc, maximum likelihood, and multiple imputation techniques. Organ. Res. Methods 6, 328–362. doi: 10.1177/1094428103254673

[ref63] Newman D. A. (2014). Missing data: Five practical guidelines. Organ. Res. Methods 17, 372–411. doi: 10.1177/1094428114548590

[ref64] Nippert-Eng C. E. (1996). Home and Work: Negotiating Boundaries Through Everyday Life. Chicago, IL: University of Chicago Press.

[ref65] Pairfam Group (2020). Codebuch Ankerperson, Welle 11 (2018/2019), Release 11.0. GESIS Data Arch. doi: 10.4232/pairfam.5678.11.0.0

[ref66] Podsakoff P. M. MacKenzie S. B. Podsakoff N. P. (2012). Sources of method bias in social science research and recommendations on how to control it. Annu. Rev. Psychol. 63, 539–569. doi: 10.1146/annurev-psych-120710-100452 21838546

[ref67] Preacher K. J. Hayes A. F. (2008). Asymptotic and resampling strategies for assessing and comparing indirect effects in multiple mediator models. Behav. Res. Methods 40, 879–891. doi: 10.3758/BRM.40.3.879, PMID: 18697684

[ref68] Preacher K. J. Rucker D. D. Hayes A. F. (2007). Addressing moderated mediation hypotheses: theory, methods, and prescriptions. Multivar. Behav. Res. 42, 185–227. doi: 10.1080/00273170701341316, PMID: 26821081

[ref69] R Core Team (2019). R: A language and environment for statistical computing. Vienna, Austria: R Foundation for Statistical Computing. Available at: www.R-project.org.

[ref70] Rosseel Y. (2012). Lavaan: an R package for structural equation modeling and more. J. Stat. Softw. 48, 1–36. doi: 10.18637/jss.v048.i02

[ref71] Rothbard N. P. Beetz A. M. Harari D. (2021). Balancing the scales: a configurational approach to work-life balance. Annu. Rev. Organ. Psychol. Organ. Behav. 8, 73–103. doi: 10.1146/annurev-orgpsych-012420-061833

[ref72] Schiffrin H. Edelman A. Falkenstern M. Stewart C. (2010). The associations among computer-mediated communication, relationships, and well-being. Cyberpsychol. Behav. Soc. Netw. 13, 299–306. doi: 10.1089/cyber.2009.0173, PMID: 20557249

[ref73] Shockley K. M. Allen T. D. (2015). Deciding between work and family: an episodic approach. Pers. Psychol. 68, 283–318. doi: 10.1111/peps.12077

[ref74] Shockley K. M. Singla N. (2011). Reconsidering work-family interactions and satisfaction: a meta-analysis. J. Manag. 37, 861–886. doi: 10.1177/0149206310394864

[ref75] Sonnentag S. Kuttler I. Fritz C. (2010). Job stressors, emotional exhaustion, and need for recovery: a multi-source study on the benefits of psychological detachment. J. Vocat. Behav. 76, 355–365. doi: 10.1016/j.jvb.2009.06.005

[ref76] Spector P. E. (1997). Job satisfaction: application, assessment, causes, and consequences. Thousand Oaks, CA: SAGE. doi: 10.4135/9781452231549

[ref77] Statistisches Bundesamt (2021). Number of divorces down 3.5% in 2020. Wiesbaden Available at: https://www.destatis.de/EN/Press/2021/08/PE21_378_126.html. Accessed April 8, 2022.

[ref78] Vaziri H. Casper W. J. Wayne J. H. Matthews R. A. (2020). Changes to the work–family interface during the COVID-19 pandemic: examining predictors and implications using latent transition analysis. J. Appl. Psychol. 105, 1073–1087. doi: 10.1037/apl0000819 32866024

[ref79] Wang B. Liu Y. Parker S. K. (2020). How does the use of information communication technology affect individuals? A work design perspective. Acad. Manag. Ann. 14, 695–725. doi: 10.5465/annals.2018.0127

[ref80] Wanous J. P. Reichers A. E. Hudy M. J. (1997). Overall job satisfaction: how good are single-item measures? J. Appl. Psychol. 82, 247–252. doi: 10.1037/0021-9010.82.2.247, PMID: 9109282

[ref81] Wolff H.-G. Höge T. (2011). Konflikte zwischen Arbeit und Familie. Z. Für Arb.-Organ. AO 55, 143–152. doi: 10.1026/0932-4089/a000053

[ref82] Wu C. Hunter E. M. Sublett L. W. (2021). Gaining affective resources for work-family enrichment: a multisource experience sampling study of micro-role transitions. J. Vocat. Behav. 125:103541. doi: 10.1016/j.jvb.2021.103541

