# Supplementary material for: Opening the doors for spillovers: a contingency view of the effects of work from home on the work–home interface
Source: Front Psychol. 2023 Jul 5;14:1191657. doi: 10.3389/fpsyg.2023.1191657 (PMC10356586; doi:10.3389/fpsyg.2023.1191657)
Supplement: Supplementary file 1 [file Data_Sheet_1.DOCX]

# Opening the Doors for Spillovers: A Contingency View on the Effects of Work From Home on the Work-Home Interface

**Appendix A: Representativeness of our sample and attrition analyses**

To understand the representativeness of our sample, we compare the age distribution with the age distribution of employed individuals from Munich (Statistisches Bundesamt, 2020). Analyses reveal that our sample is very diverse, representing all age groups (Figure A). Older employees are overrepresented (χ^2^ [6] = 81.71, *p* = .000). Females are also overrepresented (55.62% in our sample relative to 47.75% in the Census data; χ^2^ [1] = 17.20, *p* = .000). 301 of the 545 individuals sampled at T1 participated in a T2 survey (55.23%). We tested for systematic sample attrition and predicted the missing values at T2 with a logistic regression, using all 24 T1 variables (Table 2). Four variables were significant at *p* < .05. Employees with higher HWC, lower income, more children under 18, and working full time were more likely to drop out at T2. Thus, our T2 sample is affected by some systematic sample attrition; because the attrition process is partially known, we can control and correct for it.

**Figure A.** *Comparison of Working Individuals from Our Sample (N = 694) with Census Data on Employed Individuals Living in Munich for Each Age Category of the Census (Statistisches Bundesamt, 2020)
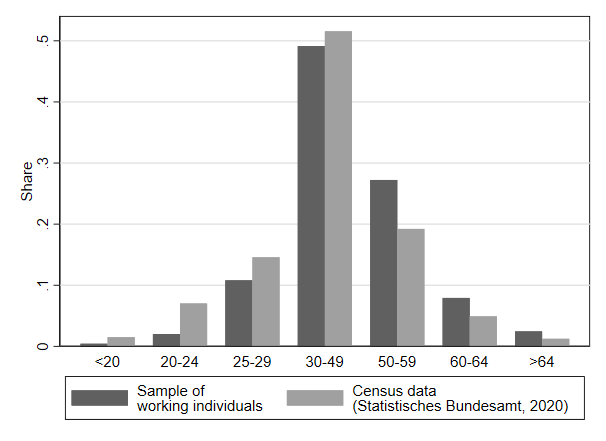
*

**Appendix B: Descriptive statistics on WFH intensity**

Figure B depicts working time patterns for employees with WFH experience before the pandemic and first-time WFH employees. The groups have parallel trends: both groups saw an increase in WFH of approximately 16 hours per week (those with WFH before the pandemic had approximately 14 WFH hours pre-pandemic, and 30 hours during the pandemic). We asked all participants who worked from home during the pandemic, “Is the Corona crisis a critical reason for why you are currently working from home?”. 415 individuals (of 540; 76%) worked from home during the pandemic, and 84% of them responded with “Yes” to this question. Of the 415 individuals, 181 did not work from home before the pandemic (99% of this group agreed to the survey question), and 234 worked from home before the pandemic (73% of this group agreed to the survey question). In summary, our measure captures WFH intensity for employees with and without prior WFH experience, which is in line with other research on WFH during the pandemic (Allen et al., 2021; Chong et al., 2020; Kossek et al., 2009; Vaziri et al., 2020).

**Figure B.** *Working Time Patterns for Experienced and First-Time WFH Employees*


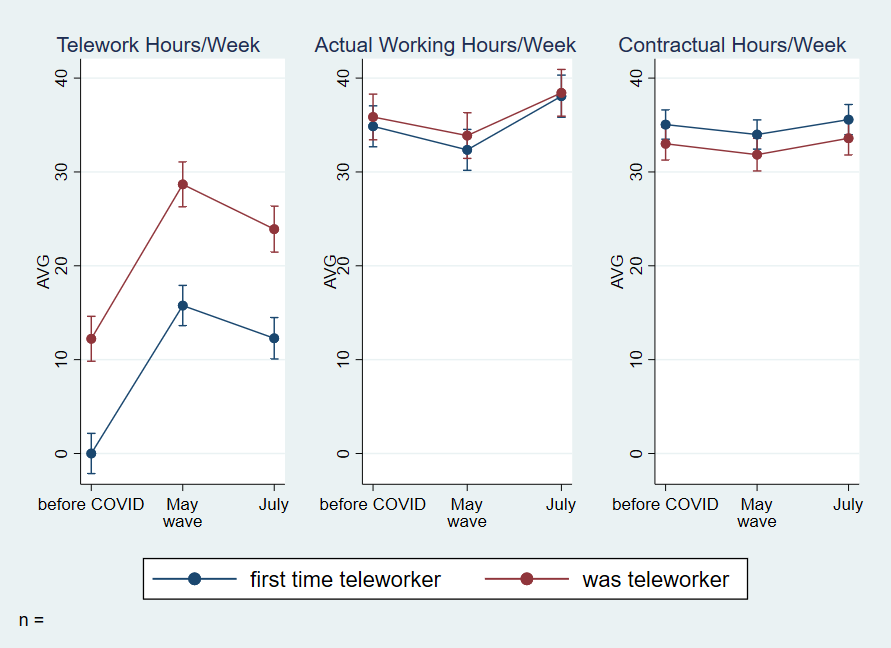


**Appendix C: Output of Figure 5**

**Table C.** *Conditional Indirect Effects of WFH Intensity on Relationship and Job Satisfaction via Work-to-Home Conflict and Home-to-Work Enrichment in a Moderated Multiple Mediation Model*

|  | Relationship satisfaction _T2_ | | | Job satisfaction _T2_ | | | | |
| --- | --- | --- | --- | --- | --- | --- | --- | --- |
|  |  | 95% CI | |  |  | | 95% CI | |
|  | Indirect effect | Lower | Upper |  | Indirect effect | Lower | | Upper |
| *Moderated mediation via WHC* |  |  |  |  |  |  | |  |
| Lower-quality equipment (-1SD) | -.010 | -.019 | -.002 |  | -.018 | -.030 | | -.008 |
| Higher-quality equipment (+1SD) | -.001 | -.006 | .004 |  | -.001 | -.009 | | .008 |
|  |  |  |  |  |  |  | |  |
| Higher vs. lower-quality | .010 | .001 | .017 |  | .017 | .005 | | .027 |
| Moderated mediation index | .004 | .001 | .008 |  | .007 | .002 | | .013 |
|  |  |  |  |  |  |  | |  |
| *Moderated mediation via HWE* |  |  |  |  |  |  | |  |
| Lower home centrality (-1SD) | .001 | -.004 | .005 |  | .000 | -.002 | | .004 |
| Higher home centrality (+1SD) | .008 | .003 | .014 |  | .006 | .001 | | .012 |
|  |  |  |  |  |  |  | |  |
| Higher vs. lower-centrality | .007 | .001 | .012 |  | .005 | .001 | | .010 |
| Moderated mediation index | .004 | .001 | .008 |  | .003 | .000 | | .006 |

Notes. *N*_T1_ = 545, *N*_T2_ = 301. Unstandardized indirect effects and Monte Carlo (20,000) confidence intervals (CI) are reported.

**Appendix D: Models with the full set of control variables**

**Table D.1.** *Direct Unconditional and Conditional Effects of WFH Intensity on Work-Home Spillovers*

| Variable | WHC | | | |  | HWC | | | | |  | WHE | | | |  | HWE | | | |
| --- | --- | --- | --- | --- | --- | --- | --- | --- | --- | --- | --- | --- | --- | --- | --- | --- | --- | --- | --- | --- |
|  | H1 | | H1a | |  | H2 | | | H2a | |  | H3 | | H3a | |  | H4 | | H4a | |
| WFH Intensity |  | .009** |  | .009** |  |  | .002 |  |  | .003 |  |  | .002 |  | .002 |  |  | .006* |  | .007* |
| Work Equipment |  |  | - | .044 |  |  |  |  |  |  |  |  |  |  |  |  |  |  |  |  |
| WFH Intensity x Work Equipment |  |  | - | .004* |  |  |  |  |  |  |  |  |  |  |  |  |  |  |  |  |
| Room Conditions |  |  |  |  |  |  |  |  | - | .02 |  |  |  |  |  |  |  |  |  |  |
| WFH Intensity x Room Conditions |  |  |  |  |  |  |  |  |  | .001 |  |  |  |  |  |  |  |  |  |  |
| Work Centrality |  |  |  |  |  |  |  |  |  |  |  |  |  |  | .116** |  |  |  |  |  |
| WFH Intensity x Work Centrality |  |  |  |  |  |  |  |  |  |  |  |  |  |  | .000 |  |  |  |  |  |
| Home Centrality |  |  |  |  |  |  |  |  |  |  |  |  |  |  |  |  |  |  |  | .128** |
| WFH Intensity x Home Centrality |  |  |  |  |  |  |  |  |  |  |  |  |  |  |  |  |  |  |  | .005* |
| Controls |  |  |  |  |  |  |  |  |  |  |  |  |  |  |  |  |  |  |  |  |
| Pre-pandemic WFH | - | .003 | - | .003 |  |  | .007† |  |  | .007† |  |  | .004 |  | .003 |  | - | .001 | - | .001 |
| Segmentation pref. |  | .203 *** | | .199*** | |  | .027 |  |  | .025 |  | - | .232*** | - | .213*** | | - | .03 | - | .044 |
| Income |  | .027 |  | .021 |  | - | .087* |  | - | .084* |  | - | .024 | - | .027 |  |  | .005 |  | .01 |
| Age | - | .008* | - | .008† |  | - | .012** |  | - | .012** |  |  | .001 |  | .001 |  | - | .011** | - | .010* |
| Female |  | .05 |  | .048 |  | - | .106 |  | - | .106 |  |  | .228** |  | .221** |  |  | .232** |  | .204* |
| Married |  | .104 |  | .116 |  |  | .205* |  |  | .209* |  |  | .066 |  | .061 |  |  | .143 |  | .128 |
| Living together |  | .123 |  | .122 |  | - | .292* |  | - | .292* |  |  | .082 |  | .063 |  |  | .121 |  | .082 |
| No. of children < 18 | - | .075 | - | .071 |  |  | .211** |  |  | .205** |  |  | .023 |  | .061 |  | - | .042 | - | .033 |
| Supervisor |  | .287** |  | .293** |  | - | .132 |  | - | .132 |  |  | .147† |  | .103 |  |  | .119 |  | .114 |
| Kurzarbeit | - | .129 | - | .119 |  | - | .121 |  | - | .132 |  | - | .008 |  | .002 |  |  | .067 |  | .053 |
| Task change |  | .187*** | | .180*** | |  | .049 |  |  | .051 |  |  | .006 |  | .000 |  | - | .002 | - | .006 |
| Household comp. |  | .005 |  | .000 |  | - | .017 |  | - | .024 |  | - | .044 | - | .083 |  | - | .228† | - | .254* |
| Industry |  |  |  |  |  |  |  |  |  |  |  |  |  |  |  |  |  |  |  |  |
| Tertiary Industry | - | .213† | - | .214† |  |  | .009 |  |  | .002 |  |  | .007 |  | .014 |  | - | .269** | - | .259** |
| Public sector | - | .264† | - | .249† |  | - | .071 |  | - | .086 |  |  | .045 |  | .058 |  | - | .188 | - | .168 |
| Contract Type: |  |  |  |  |  |  |  |  |  |  |  |  |  |  |  |  |  |  |  |  |
| Self-employed |  | .002 |  | .013 |  | - | .06 |  | - | .056 |  |  | .139 |  | .11 |  |  | .158 |  | .15 |
| Other |  | .078 |  | .126 |  |  | .505 |  |  | .511 |  | - | .111 | - | .137 |  | - | .062 | - | .07 |
| Part-time | - | .125 | - | .138 |  |  | .216† |  |  | .221† |  |  | .107 |  | .111 |  |  | .134 |  | .132 |

Notes. *N* = 545. † *p* < .10, * *p* < .05, ** *p* < .01, *** *p* < .001. Unstandardized regression coefficients. WFH intensity and all moderators mean-centered. WHC = work-to-home conflict, HWE = home-to-work enrichment; HWC and WHE accordingly.

**Table D.2**. Multiple Mediation Model of WHC and HWE on Satisfaction Outcomes Measured After 8 Weeks

|  | WHC | HWE | Rel. sat. _T2_ | Job sat. _T2_ |
| --- | --- | --- | --- | --- |
| Control variables |  |  |  |  |
| Pre-pandemic WFH | -.003 (.005) | -.001 (.004) | .016 (.013) | -.027† (.016) |
| Segmentation pref. | .210*** (.051) | -.035 (.042) | .068 (.138) | -.279† (.148) |
| Income | .027 (.048) | .004 (.049) | .055 (.130) | .190 (.178) |
| Age | -.009* (.004) | -.011** (.004) | -.010 (.012) | -.003 (.014) |
| Female | .044 (.097) | .232* (.090) | .037 (.266) | .244 (.318) |
| Married | .102 (.112) | .148 (.104) | -.042 (.317) | .690* (.310) |
| Living together | .140 (.139) | .122 (.110) | .592 (.454) | .421 (.499) |
| No. of children < 18 | -.078 (.078) | -.040 (.066) | -.353 (.270) | .015 (.258) |
| Supervisor | .294** (.110) | .120 (.107) | .416 (.344) | .365 (.330) |
| Kurzarbeit | -.124 (.124) | .064 (.114) | -.824† (.446) | -.400 (.483) |
| Task change | .188*** (.045) | -.003 (.039) | -.120 (.137) | -.024 (.162) |
| Household composition | .023 (.133) | -.231† (.119) | -.064 (.410) | .015 (.449) |
| Industry |  |  |  |  |
| Tertiary Industry | -.211 (.130) | -.274** (.096) | .491 (.432) | -.179 (.440) |
| Public sector | -.263† (.148) | -.193 (.121) | .523 (.430) | .271 (.466) |
| Contract Type: |  |  |  |  |
| Self-employed | .004 (.155) | .157 (.136) | -.371 (.501) | .065 (.534) |
| Other | .086 (.237) | -.082 (.234) | -.798 (.647) | .062 (.599) |
| Part-time | -.132 (.142) | .135 (.117) | -.420 (.361) | .133 (.424) |
| Direct effect |  |  |  |  |
| WFH Intensity | .009** (.003) | .006* (.003) | -.009 (.008) | .010 (.01) |
| Mediators |  |  |  |  |
| WHC |  |  | -.570* (.290) | -.937** (.312) |
| WHE |  |  | .723*** (.156) | .423* (.182) |
| Indirect effects |  |  |  |  |
| WHC [CI] |  |  | -.005 [-.013, .0001] | -.008 [-.018, -.002] |
| WHE [CI] |  |  | .005 [.001, .009] | .003 [.0001, .007] |
| *R^2^* | .225 | .091 | .205 | .214 |

Notes. *N*_T1_ = 545, *N*_T2_ = 301. † *p* < 0.10, * *p* < 0.05, ** *p* < 0.01, *** *p* < 0.001. Unstandardized effect sizes. Robust standard errors shown in parentheses and clustered at the couple level; Monte Carlo (20,000) CIs reported. CI = confidence interval; WHE = work-to-home enrichment, HWC = home-to-work conflict. Baseline industry category is primary/secondary industry. We include the covariance between rel. sat. and job sat., which is .902* (.361).

**Appendix E: Complete model with all possible moderations**

**Table E.1.** *Direct Conditional Effects of WFH Intensity on Negative Spillovers*

| Variable | WHC | | | | | | | | | |  | HWC | | | | | | | | |
| --- | --- | --- | --- | --- | --- | --- | --- | --- | --- | --- | --- | --- | --- | --- | --- | --- | --- | --- | --- | --- |
| WFH Intensity |  | .009** |  | 009** |  |  | .007** |  |  | .008** |  | - | .001 |  | .000 |  |  | .000 |  | .000 |
| Work Equipment | - | .062 |  |  |  |  |  |  |  |  |  |  | .023 |  |  |  |  |  |  |  |
| WFH Intensity x Work Equipment | - | .005* |  |  |  |  |  |  |  |  |  |  | .000 |  |  |  |  |  |  |  |
| Room Conditions |  |  | - | .024 |  |  |  |  |  |  |  |  |  | - | .051* |  |  |  |  |  |
| WFH Intensity x Room Conditions |  |  | - | .002 |  |  |  |  |  |  |  |  |  |  | .001 |  |  |  |  |  |
| Work Centrality |  |  |  |  |  |  | .370*** |  |  |  |  |  |  |  |  |  | - | .161** |  |  |
| WFH Intensity x Work Centrality |  |  |  |  |  | - | .001 |  |  |  |  |  |  |  |  |  |  | .001 |  |  |
| Home Centrality |  |  |  |  |  |  |  |  | - | .122* |  |  |  |  |  |  |  |  |  | .146** |
| WFH Intensity x Home Centrality |  |  |  |  |  |  |  |  |  | .002 |  |  |  |  |  |  |  |  | - | .000 |

Notes. *N* = 545. † *p* < .10, * *p* < .05, ** *p* < .01, *** *p* < .001. Unstandardized regression coefficients. Robust standard errors clustered at the couple level. WFH intensity and all moderators mean-centered. Full models including all of the control variables are in the Appendix (Table D.1). WHC = work-to-home conflict, HWE = home-to-work enrichment, HWC and WHE accordingly.

**Table E.2.** *Direct Conditional Effects of WFH Intensity on Positive Spillovers*

| Variable | WHE | | | | | | | | | |  | HWE | | | | | | | | | |
| --- | --- | --- | --- | --- | --- | --- | --- | --- | --- | --- | --- | --- | --- | --- | --- | --- | --- | --- | --- | --- | --- |
| WFH Intensity |  | .001 |  | .000 |  |  | .000 |  |  | .001 |  |  | .005*** |  | .005* |  | |  | .005* |  | .006** |
| Work Equipment |  | .075* |  |  |  |  |  |  |  |  |  |  | .021 |  |  |  | |  |  |  |  |
| WFH Intensity x Work Equipment |  | .002 |  |  |  |  |  |  |  |  |  | - | .001 |  |  |  | |  |  |  |  |
| Room Conditions |  |  |  | .060** |  |  |  |  |  |  |  |  |  |  | .008 |  | |  |  |  |  |
| WFH Intensity x Room Conditions |  |  |  | .001 |  |  |  |  |  |  |  |  |  |  | .000 |  | |  |  |  |  |
| Work Centrality |  |  |  |  |  |  | .188*** |  |  |  |  |  |  |  |  | |  | - | .024 |  |  |
| WFH Intensity x Work Centrality |  |  |  |  |  |  | .000 |  |  |  |  |  |  |  |  |  | |  | .000 |  |  |
| Home Centrality |  |  |  |  |  |  |  |  | - | .030 |  |  |  |  |  |  | |  |  |  | .158*** |
| WFH Intensity x Home Centrality |  |  |  |  |  |  |  |  | - | .002 |  |  |  |  |  |  | |  |  |  | .006** |

Notes. *N* = 545. † *p* < .10, * *p* < .05, ** *p* < .01, *** *p* < .001. Unstandardized regression coefficients. Robust standard errors shown in parentheses and clustered at the couple level. WFH intensity and all moderators mean-centered. Full models including all of the control variables are in the Appendix (Table D.1). WHC = work-to-home conflict, HWE = home-to-work enrichment, HWC and WHE accordingly.
